# Red wine consumption, coronary calcification, and long-term clinical evolution

**DOI:** 10.1590/1414-431X20187703

**Published:** 2018-11-29

**Authors:** P.L. da Luz, D. Favarato, E.H. Moriguchi, W. de Carli, N. Bruscato, R.I. Mochiduky, P. Schwartzman, C.E. Rochitte, F.R. Laurindo

**Affiliations:** 1 Instituto do Coração, Hospital das Clínicas, Faculdade de Medicina, Universidade de São Paulo, São Paulo, SP, Brasil; 2 Hospital de Clínicas de Porto Alegre, Universidade Federal do Rio Grande do Sul, Porto Alegre, RS, Brasil; 3 Associação Veranense de Assistência em Saúde, Veranópolis, RS, Brasil; 4 Hospital Moínhos de Vento, Porto Alegre, RS, Brasil

**Keywords:** Coronary calcification, Red wine, Clinical outcome, Prevention, Plaque

## Abstract

Coronary artery calcification (CAC) is associated with atherosclerotic complications. However, elevated CAC may not always imply a worse prognosis. Herein, we report the clinical evolution of long-term red wine (RW) drinkers in relation to CAC. We followed 200 healthy male habitual RW drinkers and compared them to 154 abstainers for a period of 5.5 years. The initial evaluation included coronary computed tomography angiography (CTA), clinical, demographics, and laboratory data. CAC was quantified by the Agatston score. The follow-up process was conducted by telephone calls and/or hospital record review. The composite end-point of total death, acute myocardial infarction (AMI), or coronary revascularization (or major adverse cardiac event - MACE) was assessed. The RW drinkers ingested 28.9±15 g of alcohol/day for 23.4±12.3 years. They had higher high-density lipoprotein and low-density lipoprotein, but lower C-reactive protein than abstainers. Age, total cholesterol, triglycerides, glucose, and liver enzymes were similar. History of diabetes was lower among drinkers, but other risk factors were similar. However, drinkers had higher CAC than abstainers; the mean value was 131.5±362 in drinkers vs 40.5±320 in abstainers (P<0.001). The median and interquartile range were 15 (0.0–131.5) in RW drinkers and 1 (0.0–40.5) in abstainers (P=0.003). During the follow-up, MACE was significantly lower in drinkers than in abstainers, despite their higher CAC. The difference was driven mainly by AMI (0 vs 6; P<0.03). Greater CAC values in this setting did not predict worse prognosis. A possible underlying mechanism is lesion calcification, which leads to plaque stabilization and less clinical events.

## Introduction

Many risk factors have been associated with higher coronary artery calcium score (CAC) (1,2) and linked to plaque burden and greater incidence of cardiovascular events (3). However, several recent reports identified situations in which arterial calcification does not indicate a worse prognosis. These include high performance athletes, calcified carotid plaques, stable angina patients, and long-term statin use (4–8). These findings raise the possibility that some patterns of coronary calcification process may be associated with protective clinical outcomes. Herein, we report our observations among long-term red wine (RW) consumers regarding coronary calcification and clinical evolution.

## Material and Methods

### Population

After signing the informed consent, participants were subjected to a complete clinical evaluation and questionnaire regarding drinking habits, diet, risk factors, and lifestyle. We pooled data from a previous publication (9) and an additional cohort of 100 drinkers and 54 controls, for a total of 200 healthy RW drinkers and 154 abstainers, all male, aged 50–70 years. Individuals who reported drinking at least one glass of RW 4–5 days/week for 5 years before the beginning of the study, were considered habitual drinkers. Table 1 shows their demographic and laboratory data.

### Coronary computed tomography angiography (CTA)

Multiple coronary CTA was done in an Aquilion One 320 scanner (Toshiba Medical Systems, Japan) using a single cardiac beat (prospective acquisition mode). This volumetric acquisition had a temporal resolution of 0.3 mm thick slices. Tube current and voltage values were adjusted based on the patient's body habitus using an automated algorithm (AIRD-3D). Calcium score image acquisition used prospective acquisition with 3 mm thick slices. The Agatston score was used for CAC quantification.

Total cholesterol, high-density lipoproteins (HDL), low-density lipoproteins (LDL), triglycerides, glucose, liver enzymes, and high-sensitivity C-reactive protein (hsCRP) were measured in plasma using conventional techniques.

### Follow-up of patients

Patient follow-up was ascertained through telephone calls by a nurse trained in clinical research procedures who contacted participants or their families. For patients who suffered a medical event, discharge hospital records were reviewed. Specific information about lifestyle, survival/death, acute myocardial infarction (AMI), and coronary revascularization (CR; either by surgery or angioplasty) were recorded. Only 10 (5.4%) participants were lost to follow-up.

### Statistical analysis

Baseline data are reported as percentage, mean or median, as required by the variable distribution. Comparisons were carried out using the chi-square test, *t*-test or Mann-Whitney test as appropriate, and the event-free survival data were compared using Kaplan-Meier curves and multivariate Cox-regression analysis.

A composite end-point of total death, AMI, and CR (or major adverse cardiac event - MACE) was considered to assess RW consumption and clinical course. Student unpaired *t*-test, Kaplan Meyer curves, and multivariate analysis by Cox regression were employed when appropriate.

## Results

Drinkers ingested an average of 28.9±15.7 g alcohol/day as RW for 23.4±12.3 years. At initial evaluation, significantly higher HDL and LDL levels were found in RW drinkers, but hsCRP levels were lower than in abstainers. Total cholesterol, triglycerides, and liver enzymes were similar. No significant difference was found in demographic data such as age, hypertension, and smoking. However, diabetes was more frequent in abstainers than in drinkers. Obesity rate and plasma glucose were slightly lower in drinkers, although the difference was not statistically significant (Table 1).

**Table 1:** Baseline clinical and laboratory parameters.

	Red wine drinkers(n=200)	Abstainers(n=154)	P
Age (years)	59.1 ± 6.6	58.0 ± 6.4	0.259
Glucose (mg/dL)	101.7 ± 18.3	106.0 ± 28.2	0.33
TC (mg/dL)	214.4 ± 38.6	195.3 ± 37.6	0.001
HDL (mg/dL)	49.6 ± 12.5	40.7 ± 9.6	<0.0001
LDL (mg/dL)	136.7 ± 32.9	125.0 ± 32.2	0.0009
TG (mg/dL)	150.5 ± 115.1	147.0 ± 99.2	0.76
CRP (mg/dL)	1.8 ± 2.6	2.8 ± 5.3	<0.0001
ALT (IU/L)	47.8 ± 16.9	45.2 ± 14.4	0.21
AST (IU/L)	25.6 ± 10.8	23.8 ± 9.0	0.23
GGT (IU/L)	54.8 ± 49.8	48.3 ± 40.2	0.21
Alkaline phosphatase (IU/L)	77.7 ± 18.0	82.9 ± 23.5	0.02
Creatinine (mg/dL)	1.0 ± 0.2	1.1 ± 0.2	0.001
Hypertension (n, %)	67 (33.5)	59 (38.3)	0.409
Diabetes mellitus (n, %)	9 (4.5)	18 (11.7)	0.02
Smoking (n, %)	12 (6.0)	10 (6.5)	1.0
Obesity (n, %)	25 (12.5)	30 (20.0)	0.07
Statins use (%)	11.5	11.6	ns

Data are reported as mean±SD or number and percentage. Student’s *t*-test or chi-squared test were used for statistical analysis. ns: not significant; TC: total cholesterol; HDL: high-density lipoproteins; LDL: low-density lipoproteins; TG: triglycerides; CRP: C-reactive protein; ALT: alanine aminotransferase; AST: aspartate transaminase; GGT: gamma-glutamyltransferase.

RW drinking was not associated with any side effects. All participants had a normal and active life. The average amount of alcohol ingested by drinkers was within the limits reported in the literature (13–15). Liver enzymes were within normal levels in both groups (Table 1), although the habit of drinking had more than 20 years on average.

**Figure 1:**
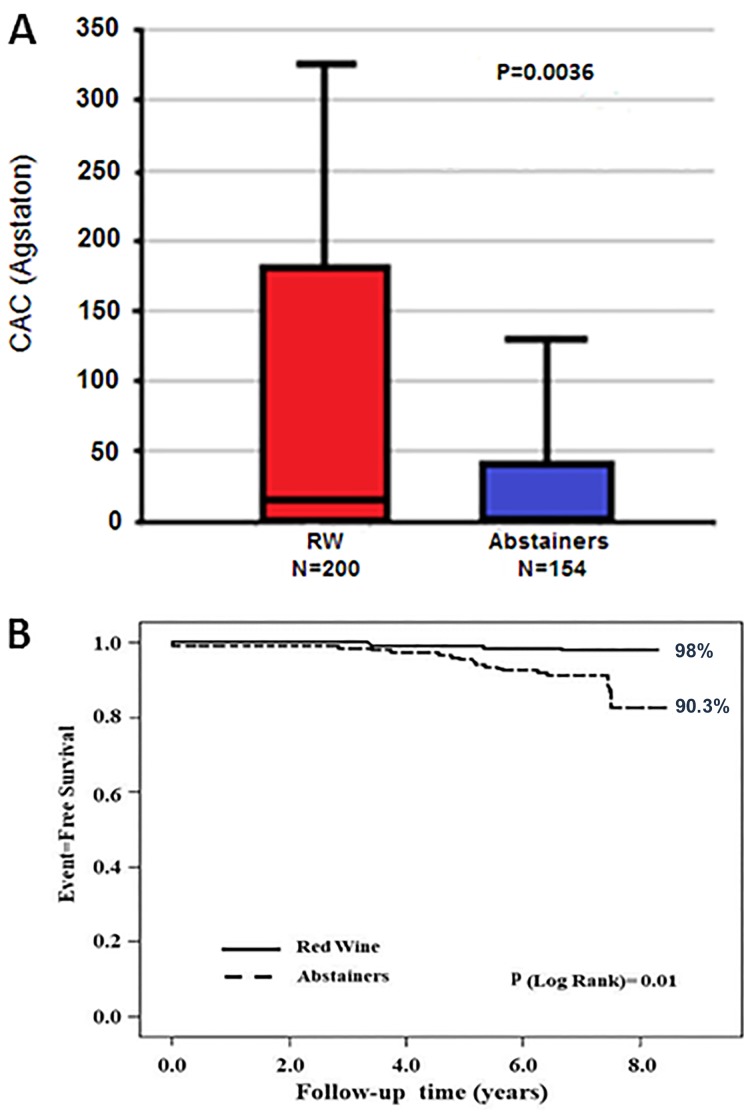
. *A*, Boxplot graphic of coronary artery calcium (CAC) scores in red wine (RW) consumers and abstainers. *B*, Event-free (death, acute myocardial infarction, revascularization) survival rates in red wine consumers vs abstainers.

CAC scores in RW drinkers were higher than in abstainers (15 (0.0–131.5) vs 1 (0.0–40.5); Figure 1A), although no significant difference was found among the total number of coronary lesions in the left main, right, left anterior descending, and circumflex coronary arteries (9). During the follow-up period of 5.5 years, the composite index of total death, AMI, and CR in drinkers was lower than in abstainers despite the higher CAC scores observed in drinkers (Figure 1B). The MACE rate per 100 person-years of follow-up was 0.2 in usual wine consumers and 1.5 in abstainers.

In order to investigate whether other variables might have interfered in the results, a multivariate analysis (Cox regression) was carried out, including wine consumption, hypertension, diabetes mellitus, smoking, and obesity. Only wine consumption and diabetes showed independent association with MACE, although in opposite directions: wine reduced events with a hazard ratio (HR) of 0.21 (95%CI: 0.068–0.644; P=0.006) and diabetes increased events with a HR of 4.2 (95%CI: 1.456–12.054; P=0.008).

Among diabetics, MACE-free survival probability in 5.5 years was 0.875 in RW drinkers and 0.722 in abstainers. Among non-diabetics, the values were 0.984 in RW drinkers and 0.841 in abstainers. Adjusted for diabetes, RW consumption was associated with better event-free survival (P=0.003; log rank; Mantel-Cox). This difference was driven mainly by a smaller incidence of AMI in drinkers than in abstainers (0 vs 6; P<0.029). Therefore, greater CAC in this setting did not predict worse prognosis; rather, it was associated with a better clinical outcome.

## Discussion

The main finding of this study was that higher CAC scores in long-term RW drinkers are not associated with worse clinical outcomes. Actually, the higher CAC scores in this setting may represent plaque stabilization and thus less clinical events. Despite the lower frequency of diabetes, slightly lower obesity rate, and plasma glucose, RW drinkers did not have lower CAC scores; instead, they had higher CAC scores. Differently, alcohol consumption was not associated with coronary calcification in the MESA study (10). However, we used a quantitative approach and MESA employed a dichotomy (absence or presence of calcium), which could explain the difference in our findings.

We also observed lower CRP in RW drinkers compared to abstainers, suggesting an anti-inflammatory effect. Several experimental and clinical findings may explain our results. Clinical studies show an inverse correlation between wine drinking and inflammation, plaque vulnerability, and arterial calcification. For instance, in carotid lesions (11), macrophage burden was greater in symptomatic than in asymptomatic plaques, which were more calcified. Beckman et al. (12) also observed that CAC in culprit lesions of acute coronary syndrome is smaller than in coronaries of patients with stable angina. Similar observations were made by Shemesh et al. (5) in AMI compared to stable angina. In parallel, RW has anti-inflammatory effects. Ingestion of RW significantly reduced MCP-1 in monocytes, as well as soluble inflammatory markers and intercellular adhesion molecules (CRP, VCAM-1, ICAM-1, and IL-1α) in healthy men (13). Hence, RW polyphenols reduce inflammation in men and may have contributed to plaque calcification in the present study. This mechanism can in part explain the RW protective effect observed in other clinical studies (14,15).

An apparent paradoxical finding was noted by Puri et al. (6). They reported coronary intravascular ultrasound findings in 3495 patients followed for 18–24 months who were treated with high-intensity statin therapy, low-intensity statin therapy, or no statin therapy. High-dose statin therapy was associated with greater calcification of coronary lesions in parallel with reduced atheroma volume compared to low-intensity statin or no statin use. Their interpretation was that statins induce plaque stabilization and hence reduce events. Even in diabetic patients, statin use increased vascular calcification, as shown in the VADT study (7). Similar results were reported by Henein et al. (8) in the Saint Francis Heart Study and EBEAT study. They documented that high-dose statin increased CAC progression over long-term follow-up. However, such increase was not associated with more events, thus suggesting that CAC progression represented plaque repair rather than expansion. Such views are shared by other authors (16,17).

Although confounding factors cannot be completely excluded, our findings are not explained by risk factors, especially diabetes and chronic renal disease, which can increase CAC (17). Regarding events, it is noteworthy that RW consumption was protective in individuals with and without diabetes. Hence, increased CAC can be reasonably ascribed to RW drinking.

Experimental studies may clarify the mechanism underlying these findings. Huang et al. (18) used finite element analysis to assess the calcification impact on mechanical stress in ruptured and stable atherosclerotic plaques and showed that ruptured lesions had a higher maximum stress. However, maximum stress was associated with increased plaque lipid content rather than calcification percentage. Studies in cultured vascular smooth muscle cells (19) also indicate that statin facilitates calcium incorporation into cells. Taken together, these considerations suggest a plausible physiological basis to explain the protective effects of some patterns of arterial calcification. These results contrast with the well-known correlation between coronary calcification and cardiovascular risk (1–3). Whether the latter reflects a distinct calcification mechanism or an earlier phase of atheroma progression deserves further study.

In the natural evolution of coronary calcification of human atherosclerosis, greater calcification implies worse prognosis. Differently, calcification of coronary arteries of RW drinkers may indicate a change in the composition of plaques, to less inflamed and more calcified. These results reinforce the notion that extensive CAC in patients subjected to treatments with an anti-inflammatory component reflect a protective phenotype of atheroma stabilization.

### Limitations and strengths

This was an observational longitudinal study and follow-up refers specifically to the clinical outcomes. Due to logistic reasons, a second CAC assessment was not possible. Specific characterization of CAC was also not possible in CTA. Thus, whether calcification was predominantly intimal or medial is unknown. However, the Agatston score used in this study is the gold standard for clinical evaluation of CAC. This long-term observation reveals a novel RW effect that deserves further exploration.
